# Being born preterm or with low weight implies a risk of infertility and premature loss of ovarian function; a national register study

**DOI:** 10.1080/03009734.2020.1770380

**Published:** 2020-06-12

**Authors:** Gunilla Sydsjö, Marie Bladh, Katarina Rindeborn, Mats Hammar, Heriberto Rodriguez-Martinez, Elizabeth Nedstrand

**Affiliations:** Faculty of Medicine and Health Sciences, Division of Obstetrics and Gynaecology, Department of Biomedical and Clinical Sciences, Linköping University, Linköping, Sweden

**Keywords:** Low birthweight, preterm birth, primary ovarian insufficiency, small for gestational age

## Abstract

**Background:** Being born with non-optimal birth characteristics has several long-term consequences on health in general but also for the individual’s reproductive pattern. In premature ovarian insufficiency (POI) the follicles are depleted or dysfunctional. This results in menopause before the age of 40, and for most of the affected women, it causes infertility. The objective of this study was to evaluate the potential effects of being born with non-optimal birth characteristics on the risk of developing POI.

**Methods:** This population-based cohort register study included all women born in Sweden between 1973 and 1993 who were followed until the end of 2012 (age at the end of follow-up ranged between 39 and 59). Women diagnosed with POI were compared with women without this diagnosis with respect to being born small for gestational age, preterm, or with low birth weight. Data on birth characteristics and diagnosis of POI were collected from national registers.

**Results:** A total of 1,033,878 women were included. Being born small for gestational age was associated with a slightly increased odds ratio of POI with 10%. Preterm birth and low birth weight were associated with somewhat increased ORs of POI after exclusion of those born small for gestational age. Similarly, being born preterm or with a low birth weight was also found to be associated with POI to the same extent.

**Conclusions:** Being born with non-optimal birth characteristics may increase the risk of premature ovarian insufficiency.

## Introduction

Being born with non-optimal birth characteristics – defined as being small for gestational age (SGA), or preterm birth, or with low birth weight (LBW) – has several long-term consequences on health in general but also for the individual’s reproductive pattern (1). Organ development during prenatal life is influenced by the intrauterine environment, and adverse conditions during foetal life can result in increased risk for metabolic syndrome, cancer, neurologic disease, and mental health problems (1). There is also evidence that young adults and adults that were born preterm and SGA have a lower chance of having children. This lowered chance of reproducing has been speculated to be due to psychological as well as medical reasons (2–4).

The medical reasons for these risks include the foetal intrauterine environment and the mother’s health and lifestyle prior to and during pregnancy (5,6). During intrauterine life, different tissues grow due to rapid cell division. Since different tissues have different timing, disturbances in the transport of nutrients, growth factors, hormones, and oxygen from the placenta may affect different tissues with different timing. The foetus adapts to growth interference by slowing down the rate of cell division (7), and undernutrition may permanently reduce the number of cells in some organs. Other effects of undernutrition include changes in the cell type distribution, hormonal feedback, metabolic activity, and organ structure due to ‘lasting’ memories of undernutrition (7). It has been shown that foetal growth restriction also affects the development of the ovaries and reproductive axis (8).

In premature ovarian insufficiency (POI) the follicles are depleted or dysfunctional, resulting in menopause before the age of 40 and for most of the affected women causing infertility (9). In a recent study, we found that POI affects 2% of all women (10). POI can be either primary or secondary, and the causes remain unknown in 90% of the cases. Even though most cases of spontaneous POI remain unexplained, the knowledge about its aetiology is increasing. Identified underlying factors include chromosomal abnormalities, gene mutations, autoimmunity, metabolic disorders, infections, and the mother’s lifestyle factors such as alcohol and nicotine use. Each of these factors can lead to either follicle dysfunction or follicle depletion (11). Iatrogenic POI is caused by surgery, chemotherapy, or radiation. Improved survival after treatment of malignant diseases in children and young adults has resulted in a rising incidence of iatrogenic POI. Even though the exact incidence is uncertain (12), it has been found that 6.3% of women treated for cancer developed POI shortly after the treatment (13). In addition, a further 8% of the study cohort developed POI later in life (12).

There is a lack of studies evaluating the potential effects of being born with non-optimal birth characteristics on the risk of developing POI. The aim of this study was to test the hypothesis that women born with non-optimal birth characteristics, that is, preterm, with low birth weight, being large for gestational age (LGA) or SGA have an increased risk of developing POI later in life.

## Material and methods

Data were collected from Swedish health data registers. The Swedish Medical Birth Register was established in 1973 and contains valid data on pregnancies, deliveries, and the new-born. The Swedish Medical Birth Register covers ∼99% of all births in Sweden (14). Data included in this study were gestational age, delivery diagnoses, and the weight and length of the infants. The National Patient Register records the in-patient care in Sweden and all specialist out-patient care. This register holds admission data, diagnoses, external causes of injury, and surgical procedures. The diagnoses in the National Patient Register are based on the Swedish version of the International Classification of Diseases (ICD). ICD-8 (version 8) was used until 1986, ICD-9 was used between 1987 and 1996, and ICD-10 has been used from 1997 and onwards. The National Patient Register has been validated for high completeness and accuracy (15–17).

In addition, we used data from the Swedish Prescribed Drug Register (18), which includes information on all prescribed and dispensed drugs in Sweden since 2005. It contains information about the patients, prescriptions, doses, amounts, costs, and prescribers’ institutions.

The study has been approved by the Regional Ethical Review Board in Linköping (03–556, 07-M66 08–08-M 233–8, 2014–112/31).

### Study design

The study population was a cohort of 1,036,918 women born in Sweden between 1973 and 1993 and were followed until 31 December 2012. Cases with POI were identified from the National Patient Register. The ICD diagnosis codes used to define POI were: 2561, 6159, 627 (ICD-8), 256.3 (ICD-9), and E28.3 (ICD-10). The personal identity number unique for each resident in Sweden allowed individual linkages of all women between the registers used. Cases with POI were also identified by dispensed systemic hormone replacement therapy for climacteric symptoms by women younger than 40 years of age. This therapy included oral and transdermal products within the ACT-groups G03CA03 oestradiol (excluding low-dose products and products for local vaginal treatment only), G03CA57 conjugated oestrogens, G03CX01 tibolone, G03FA01 norethisterone and oestrogen, G03FA12 medroxyprogesterone and oestrogen, G03FA15 dienogest and oestrogen, G03FA17 drospirenone and oestrogen, G03FB05 norethisterone and oestrogen, G03FB06 medroxyprogesterone and oestrogen, and G03FB09 levonorgestrel and oestrogen. Since these drugs are mainly prescribed to treat menopausal women and cannot be used for contraception, women prescribed these drugs who were younger than 40 years of age were considered to have POI. However, since hormone replacement therapy is also prescribed to patients with iatrogenic POI (e.g. after oophorectomy), all women with a cancer diagnosis were excluded if diagnosed with a spontaneous POI. These women were identified in the National Patient Registry using the following ICD-codes: all diagnoses starting with C in ICD-10, codes 140–239 in ICD-9, and codes 140–239 in ICD-8. Furthermore, women who had had a bilateral oophorectomy (ICD-10 code LAF10) and been prescribed and dispensed hormone replacement therapy were determined to have POI and were thus included in the study (*n* = 302). Furthermore, women having the diagnoses Turner’s syndrome (Q960-Q969), ovarian cancer (C569), and/or thyroid disease (E210-E213, E060-E069) were excluded (*n* = 3040) leaving 1,033,878 women in the final study population.

### Exposure variables and definitions

Infants born preterm were separated from those born at term to assess whether there were any differences in the incidence of POI between these two groups. Among those born preterm we further separated those born SGA from those born appropriate for gestational age (AGA). Furthermore, we categorized study infants into quartiles regarding the severity of SGA to investigate potential dose-response associations. SGA was defined as birth weight ≤2 standard deviations (SD) of the mean weight at a specific gestational age according to the Swedish standard. Large for gestational age (LGA) was defined in a similar manner but with the limit >2 SD of the mean weight for gestational age. LBW was defined as birth weight below 2500 g and very low birth weight as a birthweight below 1500 g. Moderate preterm birth was defined as being born between gestational weeks 32 and 36, and very preterm birth as being born before gestational week 32.

### Statistical analysis

Pearson’s chi-square statistic was used to explore the bivariate association between POI and socio-demographic factors and birth characteristics. To investigate whether being born preterm, with low birth weight, SGA, or LGA was related to an increased risk of POI later in life, we analyzed the data using a single and multiple logistic regression, providing odds ratios (ORs) with 95% confidence intervals (CIs). In the multiple logistic regression models, data were adjusted for attained educational level (categorized into three groups: elementary, high school, and university) among the study participants and year of birth. Each birth characteristic was modelled separately. A *p*-value <0.05 was considered statistically significant. The Mantel–Haenszel test for trend for the different levels of SGA was used. All statistical analyses were performed using IBM SPSS, version 24.0 (IBM SPSS Inc., Armonk, NY).

## Results

A total of 1,033,878 women born between 1973 and 1993 were included in the final study cohort, and 18,627 (1.8%) of these women had POI.

### Preterm delivery and risk of primary ovarian insufficiency

The cohort included a total of 51,924 women born preterm, of whom 960 (1.8%) had POI. That was identical to the proportion of women with POI among women born at term. Also, 5.0% of the women not diagnosed with POI were born preterm, while the corresponding number for women diagnosed with POI was 5.2% (*p* = 0.407) (Table 1). The unadjusted OR was not significantly increased (HR 1.03, 95% CI: 0.96–1.12) (Table 2). However, when adjusted for year of birth and educational level, being born preterm was associated with 11% increased risk of POI (OR 1.11, 95% CI: 1.04–1.19) (Table 2). When excluding women born SGA in the preterm group, the statistical differences remained (OR 1.08, 95% CI: 1.01–1.16) (Table 2).

**Table 1.** Socio-demographic and birth characteristics of the study population by the presence of primary ovarian insufficiency (primary ovarian insufficiency, *n* = 18,627).

	No primary ovarian insufficiency		Primary ovarian insufficiency		
	*n*	%	*n*	%	*p*-value
Year of birth					<0.001
1973–1975	235,118	23.2	7977	42.8	
1978–1982	218,071	21.5	5050	27.1	
1983–1987	226,642	22.3	3225	17.3	
1988–1993	335,420	33.0	2375	12.8	
Education					<0.001
Elementary	67,560	6.8	991	5.4	
High school	442,870	44.9	6632	35.8	
University	476,811	48.3	10,889	58.8	
Missing	28,010	2.8	115	0.6	
Gestational age					<0.001
Very preterm	4968	0.5	96	0.5	
Preterm	45,996	4.5	864	4.6	
Term	871,826	85.9	15,652	84.0	
Post term	92,461	9.1	2015	10.8	
Preterm birth					0.407
No	964,287	95.0	17,667	94.8	
Yes	50,964	5.0	960	5.2	
Birthweight					<0.001
Very low birthweight	4655	0.5	91	0.5	
Low birthweight	38,557	3.8	783	4.2	
Normal birthweight	972,039	95.7	17,753	95.3	
Low birthweight					<0.001
No	972,039	95.7	17,753	95.3	
Yes	43,212	4.3	874	4.7	
Small for gestational age (1SD)					<0.001
No	808,140	79.6	14,354	77.1	
Yes	207,111	20.4	4273	22.9	
Small for gestational age (2SD)					<0.001
No	977,074	96.2	17,781	95.5	
Yes	38,177	3.8	846	4.5	
Small for gestational age (3SD)					0.006
No	1,009,542	99.4	18,494	99.3	
Yes	5709	0.6	133	0.7	
Large for gestational age (1SD)					<0.001
No	878,038	86.5	16,374	87.9	
Yes	137,213	13.5	2253	12.1	
Large for gestational age (2SD)					0.076
No	987,461	97.3	18,157	97.5	
Yes	27,790	2.7	470	2.1	
Large for gestational age (3SD)					0.436
No	1,010,548	99.5	18,548	99.6	
Yes	4703	0.5	79	0.4	
Delivery method					0.057
Normal	859,936	84.7	15,896	85.3	
Instrumental	52,227	5.1	915	4.9	
Section	103,088	10.2	1816	9.7	

**Table 2.** Crude and adjusted odds ratios and corresponding 95% confidence intervals (CIs) of primary ovarian insufficiency among infants born with different characteristics.

	Crude OR	95% CI	*p*-Value	Adjusted OR^a^	95% CI	*p*-value
Premature delivery (<37 gestational weeks)	1.03	0.96–1.12	0.407	1.11	1.04–1.19	0.001
Premature delivery, excluding SGA	1.00	0.93–1.07	0.997	1.08	1.01–1.16	0.037
Low birthweight (<2500 g)	1.11	1.03–1.19	0.004	1.15	1.07–1.23	<0.001
Low birthweight, excluding SGA	1.02	0.92–1.12	0.715	1.10	1.00–1.22	0.053
Small for gestational age (1SD)	1.16	1.12–1.20	<0.001	1.05	1.02–1.09	0.004
Small for gestational age (2SD)	1.22	1.14–1.31	<0.001	1.10	1.02–1.18	0.009
Small for gestational age (3SD)	1.27	1.07–1.51	0.006	1.16	1.07–1.38	0.095
Large for gestational age (1SD)	0.88	0.84–0.92	<0.001	0.95	0.91–1.00	0.036
Large for gestational age (2SD)	0.92	0.84–1.01	0.076	1.00	0.91–1.09	0.908
Large for gestational age (3SD)	0.92	0.73–1.14	0.436	0.96	0.76–1.20	0.707
SGA, excluding PT and LBW	1.22	1.11–1.35	<0.001	1.01	0.91–1.12	0.841
Preterm, excluding SGA and LBW	0.98	0.89–1.08	0.663	1.05	0.95–1.15	0.321
LBW, excluding SGA and preterm	0.97	0.75–1.24	0.803	1.03	0.80–1.32	0.843

^a^Adjusted for year of birth and educational level.

### Low birth weight and risk of primary ovarian insufficiency

A total of 44,087 women were born with low birth weight, and 952 (2.0%) of them had POI compared with 1.8% in women born with normal birth weight. Additionally, 4.7% of the women diagnosed with POI were born with a low birth weight compared with 4.3% among the women born with normal birth weight (*p* ≤ 0.1) (Table 1). LBW was associated with a 15% increased risk of POI in the adjusted analysis (OR 1.15, 95% CI: 1.07–1.23). When women born SGA were excluded, the difference remained significant though the risk was lower (OR 1.10, 95% CI: 1.00–1.22) (Table 2).

### Small for gestational age and risk of primary ovarian insufficiency

Among 211,362 women born SGA, with a weight less than 1 SD below the mean (1SD), 39,023 women were born 2 SD below the mean (2SD), and 5,842 were born 3 SD below the mean (3SD) (Table 1). Being born SGA (1SD) was associated with a 5% increased risk of POI (OR 1.05, 95% CI: 1.02–1.09). Being born SGA (2SD) indicated a 10% increased risk of POI (OR 1.10, 95% CI: 1.02–1.18). Finally, being born SGA (3SD) was associated with a 16% increased risk of POI (OR 1.16, 95% CI: 1.07–1.38) (Table 2). Using Mantel–Haenszel’s test for trend, the likelihood of being diagnosed with POI increases with the severity of the growth restriction (*p* < 0.001, data not shown).

### Large for gestational age and risk of primary ovarian insufficiency

Being born LGA was not associated with the risk of developing POI (Table 2).

## Discussion

This study found an association between being born with non-optimal birth characteristics and risk of POI later in life. Being born in SGA, preterm, or LBW was associated with a somewhat increased likelihood of being diagnosed with POI.

To our knowledge, there are no published studies investigating the relation between non-optimal birth characteristics and risk of POI. However, an association of being born LBW or SGA with the risk of infertility has been shown (19,20). Also, women born with low birth weight have been found to be younger when reaching natural menopause (21). The reason for this is unknown, but intrauterine growth restriction has been related to smaller internal genitalia, reduced ovulation rate, and elevated FSH levels in women (8,22,23).

The increased risk of POI among individuals born SGA and LBW may be due to the influence of under-nutrition during the intrauterine life, which in turn affects the development or survival of the follicles. This might lead to dysfunction of the follicles or lower numbers of follicles, resulting in an increased likelihood of developing POI later in life. Preterm birth might not affect the follicles since the follicles usually develop normally if no under-nutrition is present. However, potential explanations for the associations between non-optimal birth characteristics (SGA, LBW, and preterm) and POI is that being born with these characteristics increases the risk of several autoimmune diseases, many of which in turn are associated with POI, for example, primary adrenal failure (Addison’s disease), autoimmune thyroid disease (Schmidt’s syndrome), and type 1 diabetes (24,25). It is thus possible that non-optimal birth characteristics lead to an increased risk of autoimmune disease, ending in autoimmune POI.

POI results in premature cessation of ovulation and infertility. This can lead to involuntary childlessness if it occurs among women who have not yet considered becoming pregnant, especially since women increasingly postpone childbearing. Since there is no medical therapy today that is proven to improve the ovarian function and fertility for women with POI, fertility preservation is the only solution if the women want children later in life (26). This must be done before the follicles are depleted, and it may be too late at the time of diagnosis. It is therefore of great importance to identify and follow women with a high risk of POI so that an early diagnosis can be made before it is too late with fertility preservation. It should be emphasized, however, that women with POI may still become pregnant.

This study has a methodological strength in using a national population-based design, the high validity and completeness of the databases, the large size of the study cohort, and the long follow-up of the study participants. A limitation is a fact that there probably are women with amenorrhoea who have not been diagnosed with POI, making it likely that we underreported the prevalence in our study. The missing of the outcome is, however, not dependent on the birth characteristics, which means that this issue would dilute associations rather than cause them. Moreover, by using POI identified not only from the National Patient Register but also from the Swedish Prescribed Drug Register, we found additional women and reduced the figure missing the diagnosis. It should be emphasized, however, that some of the POI cases identified by means of the Swedish Prescribed Drug Register maybe women prescribed hormonal therapy due to causes other than POI, such as in conjunction with the treatment of infertility. Another limitation is the lack of some potential confounding factors for which we did not have data, for example, body mass index, smoking or alcohol consumption, or year of diagnosis in the Swedish Prescribed Drug Register. However, these factors probably do not cause POI (5) but may affect the timing of the amenorrhoea. We did, however, adjust for some other factors, that is, educational level, and year of birth, which might be more relevant to control for. Still, the findings in the current study should be interpreted with caution since it is possible that some of the associations found could be affected by factors not included in the analyses. Hence, further studies are needed to validate and to deepen the knowledge on the causes of being diagnosed with POI.

In conclusion, this population-based and nationwide Swedish cohort study show that being born with non-optimal birth characteristics was associated with a slightly increased risk of POI. These findings not only provide new knowledge of the origin of POI but can also help identify women at an increased risk of POI, who should be informed about the risks and the consequences of POI. An implication is that women born SGA may be advised not to postpone their pregnancies too long.
